# Macular Inner Retinal Layer Thinning in Diabetic Patients without Retinopathy Measured by Spectral Domain Optical Coherence Tomography

**Published:** 2018

**Authors:** Mário Pincelli Netto, Verônica Castro Lima, Maria Angélica Pacheco, Nichard Unonius, Carolina PB. Gracitelli, Tiago Santos Prata

**Affiliations:** 1 Department of Ophthalmology and Visual Sciences, Federal University of São Paulo, São Paulo, Brazil; 2 Hospital Medicina dos Olhos, São Paulo, Brazil

**Keywords:** Spectral Domain Optical Coherence, Diabetes, Macular Inner Retinal Layer Thickness, Diabetic Retinopathy

## Abstract

The aim of this study was to use Spectral Domain-Optical Coherence Tomography (SD-OCT) to measure the thickness of the Macular Inner Retinal Layer (MIRL) and compare the results between diabetic patients with no signs of retinopathy and healthy subjects.

Overall, 47 type 2 diabetic patients without clinical signs of retinopathy were prospectively analyzed along with 36 healthy subjects. This study excluded patients with other systemic or ocular diseases. All patients had their MIRL thickness measured by RTVue-100 SD-OCT (7x7 mm macular grid). The MIRL thickness is provided by the ganglion cell complex scan (comprised of the retinal nerve fiber, ganglion cell, and inner plexiform layers). Only one eye was randomly selected if both were eligible for analysis.

Mean age was similar between the two groups (diabetic patients: 57.3 ± 10.6 and control subjects: 60.2 ± 12.2 years) (P = 0.19). No significant differences regarding optic disc area and cup-to-disc ratio was observed in the comparison of the two groups (P ≥ 0.38 for both comparisons). In patients with diabetes, the average MIRL was significantly thinner when compared to controls (91.6 versus 96.2 micrometer (µm); P = 0.02). Regional analysis revealed superior and inferior MIRL to be significantly thinner in patients with diabetes than the controls (P ≤ 0.04). The juxtafoveal area was compromised (thinned) in 70% of diabetic eyes, classified as abnormal (P < 1%; compared to the device’s normative database).

In conclusion, patients with type 2 diabetes without clinical evidence of retinopathy had lower MIRL average values when compared to the control group. This can be explained by the ischemia and retinal tissue injury caused by diabetes even in early stages of diabetic retinopathy, which can affect MIRL thickness. Possible implications of these findings on diagnosis and treatment of diabetic retinopathy requires further investigation.

## INTRODUCTION

Diabetic Retinopathy (DR) is one of the most important causes of blindness, worldwide [1, 2]. It is characterized by alterations of the retinal microvasculature and involves endothelial cells and pericytes loss [1, 2]. Resulting features include microaneurysms, hemorrhages, capillary non-perfusion, and neovascularization [1, 2]. There is evidence suggesting that functional impairment usually precedes the earliest clinical manifestations of diabetic retinal vasculopathy. Focal Retinal Nerve Fiber Layer (RNFL) loss in diabetic patients with preclinical DR, detected by red-free fundus photograph, was first described by Chihara et al. [3]. Later, Lopes de Faria et al. found localized RNFL loss in patients with type 1 diabetes (T1DM) without signs of retinopathy, by scanning laser polarimetry [4]. Recently, Kim et al. found that patients with DR progression exhibited, among other systemic changes, longer Type 2 Diabetes Mellitus (T2DM) duration, thinner macular Ganglion Cell-Inner Plexiform Layer (mGCIPL), and greater mGCIPL thinning rate [5]. Also, in vitro studies have demonstrated that diabetes influences both retinal neurons and glial cells; Retinal Ganglion Cells (RGCs) apoptosis was observed even before the onset of retinopathy [6, 7].

Optical Coherence Tomography (OCT) is a tool that allows quantitative measurements of retinal thickness [8-10]. It provides cross-sectional images of the retina and useful information on vitreoretinal changes associated with a high diversity of ocular diseases [8-10]. Spectral-Domain OCT (SD-OCT) is one of the latest generations of techniques and provides images with high axial resolution and quality [11-13]. In addition, it allows identification of individual retinal layers approaching histological detail with significant information in several diseases [14-17]. The Ganglion Cell Complex (GCC) scan was designed to improve glaucoma diagnosis and is composed of the inner three layers of the retina (RNFL; ganglion cell layer and inner plexiform layer) [16, 18, 19].

Although retinopathy is a long-known complication of diabetes, the relationship between RNFL thickness and diabetes in the early stage of the disease has only recently been established. Tekin et al. compared children with Type 1 DM (T1DM) without retinopathy with a control group, and suggested that retinal neural changes, shown by SD-OCT, could be present in diabetic eyes, even before clinically detectable retinal vasculopathy [20]. On the other hand, Pekel et al. did not find any differences in the RNFL thickness of children with T1DM, yet found that binocular RNFL thickness symmetry percentage was significantly different in diabetic and control groups [21]. Other groups also corroborated that neurodegeneration by T2DM may play a role in the thinning of GCC, using the SD-OCT [20, 22-28]. Considering the mentioned points, the current authors hypothesized that even in the early stage of the disease, diabetes leads to ischemia and retinal tissue injury, affecting Macular Inner Retinal Layer (MIRL) thickness. Therefore, this study aimed at comparing values of MIRL thickness obtained by a SD-OCT device between diabetic patients without clinical signs of retinopathy and healthy subjects.

## MATERIALS AND METHODS

A prospective study was carried out. The protocol adhered to the tenets of the Declaration of Helsinki and was approved by the Institutional Review Board. In addition, written informed consent was obtained from all subjects.

**Patients**

Diabetic patients without clinical signs of retinopathy and healthy subjects were consecutively enrolled in this study. Patients with minimal visible changes of diabetic retinopathy (such as microaneurysms, macular edema, macular, macular thickening, or any sign of diabetic retinopathy), other ocular diseases, media opacities (excluding mild cataract), history of ocular trauma or surgery, refractive error greater than ± 5 Diopter (D), spherical or cylindrical corneal thickness greater than ± 3D, and central corneal thickness (based on ultrasound pachymetry) above 600 micrometer (µm) or below 450 µm were excluded. Eyes with glaucoma were excluded based on IOP ≥ 21 mmHg and clinical signs of neuropathy or visual field suggestive findings. Baseline data included age, gender, race, presence of T2DM, and time of diagnosis (based on the information provided by patients). Two masked ophthalmologists classified the patients and when there was any disagreement, a third ophthalmologist was recruited. Therefore, when no criteria for DR was found, the patient was classified as “no retinopathy” and if any criteria for DR (based on ETDRS) was found in the clinical examination, these patients were excluded [26-28].

Diabetes was defined according to self-reported physician diagnosis, and all patients with diabetes were under medical treatment within at least six months (using only oral hypoglycemic agents). Healthy subjects were recruited from the general population or from spouses and relatives of patients with diabetes. They were defined as having self-reported history of normal glucose level in the past two years.

**Procedures**

All participants underwent a complete ophthalmic evaluation, including slit lamp biomicroscopic examination, Goldman applanation tonometry, and fundoscopy with pupil dilation. Fundus photography was performed for all patients. Grading of DR and presence of diabetic macular edema were based on the International Clinical Diabetic Retinopathy Disease Severity Scale [29]. Analysis of MIRL thickness was performed using SD-OCT (GCC scan; RTVue-100 OCT; Optovue, Inc., Fremont, CA). The GCC scan consisted of 15 vertical line scans covering a seven-square millimeter (mm²) region. It was centered at 1-mm temporal to the fovea center for better coverage of the temporal region. The GCC scan also included a horizontal line scan at the middle for registration of vertical scans and fovea center searching. In total, it captured 15.000 data points within 0.6 seconds. The OCT scans are processed automatically to provide a thickness map of the GCC, which is defined by the distance from the internal limiting membrane to outer inner plexiform layer and is composed of the inner three layers of the retina. Some parameters of the optic nerve head report were used for analysis and included the optic disc area and cup-to-disc ratio. For this study, images with signal strength index less than 40 or those that were not well-centered (subjective assessment) were excluded from the analysis [30].

**Statistical Analysis**

Descriptive statistics included mean and standard deviation (SD) values for normally distributed variables. This study used Skewness/Kurtosis tests and histograms to check for normality. Independent samples T-test was used to compare different SD-OCT parameters between groups and other continuous variables. Categorical data were compared using the chi-square test. Whenever both eyes were eligible, one was randomly selected for analysis. All statistical analyses were performed with a commercially available software, Medcalc version 7.4.2.0 (Medcalc Software, Mariakerke, Belgium). The α level (type I error) was set at 0.05.

## RESULTS

A total of 36 healthy subjects (36 eyes) and 47 patients with diabetes (47 eyes) were enrolled. Table 1 shows baseline characteristics of the groups, including age, gender, race, and mean values of disc size and vertical cup-to-disc ratio. Mean ± SD of age was similar between patients with diabetes and control subjects (57.3 ± 10.6 and 60.2 ± 12.2 years, respectively; P = 0.19). There were no significant differences regarding optic disc area (P = 0.59) and vertical cup-to-disc ratio between groups (P = 0.38).

**Table 1:** **Demographic characteristics of the studied subjects**

Variables	Control Group (n = 27)	Diabetic Group (n = 38)	P value
Age (years) (Mean ± SD)	60.3 ± 12.4	56.3 ± 10.8	0.18
Gender (F/M) (n)	12/15	25/13	0.14
Race (C/O) (n)	16/11	27/11	0.46
Optic Disc Size (mm^2^) (Mean ± SD)	1.94 ± 0.47	2.02 ± 0.6	0.59
C/D Ratio (Mean ± SD)	0.38 ± 0.16	0.34 ± 0.17	0.38

F: female; M: male; C: Caucasian; O: others; C/D: cup-to-disc; n: number; SD: standard deviation

Data in table are presented as mean ± SD.

**Figure 1:**
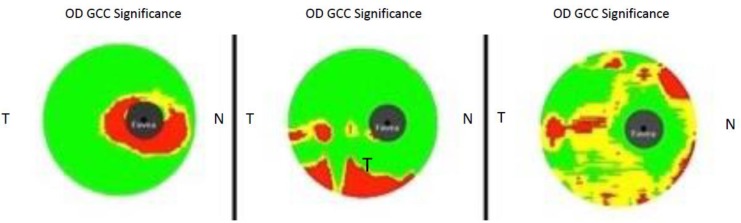
Ganglion Cell Complex (GCC) Analysis Results from Three Different Patients with Diabetes Included in the Current Study.

These Three Cases represent Three Different Patterns of GCC Thinning. (A) Perifoveal Thinning; (B) Peripheral Thinning; and (C) Diffuse Thinning.

(OD, Right Eye; T, Temporal; N, Nasal)

Average MIRL was significantly thinner in patients with diabetes compared to controls (91.6 versus 96.2 µm; P = 0.02). Additionally, regional analysis revealed superior (91.5 vs 96.3 µm; P = 0.02) and inferior mean values (91.7 versus 96.1 µm; P = 0.04) to be significantly thinner in patients with diabetes compared with controls. Although a greater difference between groups was found in the superior macular region (-4.8 µm) when compared to the inferior macular region (-4.4 µm), this difference was not statistically significant (P = 0.35).

Finally, 70% of all diabetic eyes, analyzed in this study, had an abnormal area on GCC significance map (reduced thickness; P < 1%), as classified by the device´s normative database. In 70% of eyes, the juxtafoveal area was the affected (thinned) one. Three cases representing different patterns of GCC thinning are provided in Figure 1.

## DISCUSSION

This research found that patients with T2DM without clinical evidence of DR have lower MIRL average values when compared to the control group. This difference appears to be more pronounced in the superior macula (although it was not statistically different), and the juxtafoveal area was more affected in most patients. Glaucoma is an optic neuropathy, characterized by loss of RGCs and their respective axons. In this context, GCC analysis was specifically designed to improve glaucoma diagnosis accuracy [31]. It has been shown that its diagnostic power is higher than total macular thickness. Additionally, the combination of faster speed (high-density scanning over a large region of the macula with less motion artifact) and higher resolution images with the SD-OCT significantly improved the precision of GCC measurements, which could improve the ability to track glaucomatous thinning over time [18].

Several studies of histological material have demonstrated that RGCs seem to be lost in diabetic patients [6, 32]. Immunohistochemical studies have shown that RGCs undergo apoptosis in humans with diabetes, leading to a reduction in the thickness of RNFL [6, 32]. Moreover, in vivo use of scanning laser polarimetry and other techniques, such as OCT, found significant thinning of the RNFL in diabetic eyes (likely to be due to loss of RGCs and their axons in this disease), even without clinical signs of retinopathy [3, 4, 33]. Functional changes, such as deficits in the electro-retinogram and reduced contrast sensitivity can occur before the gross vascular defects become clinically detectable [34, 35]. Some studies have investigated early changes of DR in the inner retina (ganglion cell layer) using OCT, and the results are consistent with the current findings [20, 22, 28]. Patients with no or minimal DR changes underwent OCT examination with automated segmentation of intra-retinal layers, using a custom-built algorithm [26]. The authors found reduced values for RNFL thickness and ganglion cell/inner plexiform layer complex thickness only in the group with mild DR [26]. In the present study, patients with no retinal abnormalities were included and they were found to have significantly reduced MIRL thickness when compared to controls. Using SD-OCT, a recent study found that superior macular GCC thicknesses were significantly decreased in diabetic cases without retinopathy, and no significant peripapillary RNFL thickness changes were observed [27]. These results are in accordance with the current findings, and prove that GCC reduction occurs much earlier than peripapillary RNFL thinning in diabetic patients without retinopathy [27].

Using a different imaging technology (scanning laser polarimetry), Lopes de Faria et al. [4] also found a significant nerve fiber layer loss in the superior segment of the retina in patients with T1DM mellitus without retinopathy. Although only patients with T2DM were included in the current study, a more pronounced thinning of the MIRL in the superior hemisphere was also found (even though this difference did not reach statistical significance). If there is an asymmetric structural loss, it is still to be determined and better understood. Another recent study used a multivariable analysis to compare The Ganglion Cell Inner Plexiform Layer (GCIPL), using the SD-OCT, between controls and T2DM with and without RNFL defects [28]. This study showed that diabetic eyes with RNFL defects had a significantly thinner average GCIPL thickness than those without RNFL defects. Also, the presence of early and definite Cardiovascular Autonomic Neuropathy (CAN) contributed significantly more to the decreased average GCIPL thickness, relative to the absence of CAN involvement. These results may be used, in the future, as markers of early neurodegenerative changes during diabetes [28].

The findings of this study corroborate with the main hypothesis that diabetic retinal neuropathy is caused by direct damage from chronic hyperglycemia, and is not the effect of vascular DR. However, the exact nature of their interdependence is not yet known. Each process, once established, probably contributes to the progression of the other. The hypothesis that diabetes may cause retinal neuropathy independent of retinopathy is peculiar and curious, and potentially links retinal neuropathy to other diabetic neuropathies [27, 36]. Neuro-retinal degeneration creates several metabolic and signaling pathways, which are involved in the microangiopathic process as well as in the disruption of the blood-retinal barrier, which is a crucial element in the pathogenesis of DR [27, 37].

It is important to mention some specific characteristics of the current study. Since this study was cross-sectional, the researchers could not evaluate the possible effects of retinopathy progression on MIRL thickness measurements. This may help better understanding of the effects of diabetes on the inner retinal layers. Moreover, the time of T2DM diagnosis was based on the information provided by patients, which is subjective and probably lacks reliability. Finally, glycemic control, which was not evaluated in the current study, might be correlated to MIRL changes and should be addressed by further studies. Furthermore, another relevant analysis that could be addressed by future studies is the evaluation of fluorescein angiography or Optical Coherence Tomography Angiography (OCTA) in diabetic patients without clinical retinopathy, which could be relevant to understand the relationship with MIRL thickness, obtained by a SD-OCT.

## CONCLUSIONS

In conclusion, T2DM patients without retinopathy have reduced average MIRL values when compared to normal subjects, even though the vascular component of DR is absent. This difference seems to be more pronounced juxtafoveally, and it could be a marker of early manifestations in diabetic patients [28]. Possible implications of these findings on diagnosis and treatment of DR require further investigation.
